# Sexual satisfaction and sexual behaviors during the COVID-19 pandemic: results from the International Sexual Health And REproductive (I-SHARE) health survey in Luxembourg

**DOI:** 10.1186/s12889-022-13509-x

**Published:** 2022-06-03

**Authors:** Vinicius Jobim Fischer, Raquel Gómez Bravo, Alice Einloft Brunnet, Kristien Michielsen, Joseph D. Tucker, Linda Campbell, Claus Vögele

**Affiliations:** 1 Research Group Self-Regulation and Health, Institute for Health and Behaviour, Department of Behavioural and Cognitive Sciences, University of Luxembourg, Esch-sur-Alzette, Luxembourg; 2 Laboratoire Clinique Psychanalyse Développement (CLIPSYD - EA4430), University of Paris Nanterre, Paris, France; 3 International Centre for Reproductive Health, Department of Public Health and Primary Care, Faculty of Medicine and Health Sciences, Ghent University, Ghent, Belgium; 4 Institute for Global Health and Infectious Diseases, University of North Carolina, Chapel Hill, USA; 5 Centre for Population, Family and Health, University of Antwerp, Antwerp, Belgium

**Keywords:** Sexual satisfaction, Sexual behaviors, Covid-19, I-SHARE, Luxembourg

## Abstract

**Aim:**

To identify the impact of COVID-19 measures on sexual behaviors and sexual satisfaction in Luxembourg residents.

**Methods:**

We conducted a cross-sectional online survey of adults (> 18 years of age) residing in Luxembourg, while COVID-19 restrictions were in place. The survey was available from January 15 to February 12, 2021 in four languages (French, German, English and Portuguese). Survey questions focused on masturbation, cuddling, condom use, sex frequency, sexting, cybersex, watching porn, and sexual satisfaction.

**Results:**

557 volunteers completed the survey (35.5% men, 64.3% women). Sexual satisfaction and sexual problems variables were assessed on 4-point Likert scales (0 = not at all/never to 3 = very/often). Sexual problems increased during the COVID-19 measures while sexual satisfaction decreased compared to before the introduction of COVID-19 restrictions (assessed retrospectively). Factors associated with increased odds of sexual satisfaction included having a steady relationship before COVID-19 restrictions, engaging in sexting, reporting good mental health and not altering alcohol intake.

**Conclusions:**

The context of the COVID-19 pandemic and the measures implemented in Luxembourg affected sexual behaviors and sexual satisfaction. Sexual and reproductive health care centers and health professionals in general should take these results into consideration when providing care. Recommendations on the importance of sexual health for general wellbeing and behaviors associated with sexual satisfaction should be offered and possibilities to experience sexuality while reducing contamination risks be discussed.

## Introduction

Since the beginning of the pandemic, the majority of governments around the world began implementing measures to interrupt the spread of SARS-CoV-2. Although varying in scale, these measures impacted people’s daily lives. These measures included mandatory use of masks, and restrictions in movement, transit, gatherings, and partial or full confinement [1].

In Luxembourg, after the identification of the first COVID-19 case on March 01, 2020, the government established a pandemic crisis unit and implemented a variety of measures to contain the spread of the virus, including night-time curfew, shutting down services and commerce not considered essential, and mandatory mask use in public spaces. By July 2020, Luxembourg received a score of 45 in the Oxford Stringency Index regarding the stringency of measures introduced to contain the virus (with 0 = no measure to 100 = high restrictions) [2]. In addition, as a national health policy measure, large-scale testing was offered to the population [3].

Luxembourg is one of the best-performing EU countries in terms of avoiding the worst of the economic impact of the COVID-19 lockdown, in comparison to other EU countries [2]. Governmental restrictions (remote working and schooling, closure of commerce, restaurants and sport facilities, nightly curfew, prohibition of social gatherings at home, among others) altered daily living drastically, with implications for individual well-being [4]. Furthermore, fear of infection or passing the virus on to relatives or friends might elicit feelings of anxiety, depression and other responses [5]. For example, an online survey conducted in six European countries in April 2020 found a marked increase in stress, anxiety and depression since the pandemic [4]. For cohabiting couples, the lack of possibilities for meeting others, and the reduction in leisure time activities likely increased the amount of time that couples spent together. This could lead to both positive (e.g., strengthening relationships, gratitude, appreciation and tolerance [6] and negative outcomes (e.g. exacerbation of quarrels, couples’ bond diminution) [7]. It is plausible for such changes to also have affected sexual behaviors and sexual satisfaction.

To date, there is limited evidence on the impact of the COVID-19 pandemic on sexual behaviors. A study from the USA found a diversification of practices but not an increase in frequency of sexual behavior [8]. There seems to have been an increase in porn watching and in uploading sexual videos from platforms in different countries [9]. Results on sexual frequency are mixed: while Li et al. [10] and Cito et al. [11] found a decrease in sex frequency among young adults in China and in Italy, respectively, Arafat et al. [12] found no change. More conclusive results on sexual frequency would be important, as the lack of sexual activity during the lockdown has been shown to be associated with an increased risk of developing anxiety and depression [13].

Considering the importance of sexual health for general health and wellbeing [14], there is a need to better understand the impact of social restrictions on individuals’ wellbeing and their sexuality. To address these and other questions around the impact of COVID-19 related lockdown measures and their effect on sexual behaviors, the I-SHARE consortium was established in early 2020. This is a collaborative effort including 30 countries with the aim of investigating sexual and reproductive health during the COVID-19 pandemic around the world [15]. The present paper presents I-SHARE results from Luxembourg. Luxembourg is one of the most densely populated countries in Europe, with almost half of the population located in the capital city of Luxembourg [16, 17].

Multilingualism and multiculturalism are two important facets of Luxembourg’s cultural identity, with the five largest foreign communities representing 32.3% of the total population. Furthermore, the workforce is strongly based on cross-border employees, with around 185,000 employees commuting to Luxembourg daily from France, Germany and Belgium.

The present study aims to examine the impact of COVID-19 governmental measures on the perception of change regarding sexual satisfaction, sexual behaviors, substance use and mental health in a sample of volunteers residing in Luxembourg. Specifically, we investigated factors associated with sexual satisfaction during COVID-19 related restrictions imposed at the time of the present survey, including nightly curfews, mandatory facemasks use in enclosed public spaces, closed restaurants, maximum host of 2 people from other household, and closed indoor sports facilities [18].

As these measures involved several social restrictions, we hypothesized that sexual behaviors would change as a result of these lockdown measures. With respect to in-person partnered sexual activities, we expected to find solo activities and online use for sexual purposes to increase due to the restrictions put in place.

## Materials and Methods

### Study population, procedure and tools

We conducted an online, cross-sectional survey from January 15 to February 12, 2021. The survey was advertised on several University of Luxembourg media platforms (website, Facebook, Instagram and Twitter) and via national agencies, focused on sexual and reproductive health. The questionnaire was available in four languages: French, German, English and Portuguese.

Prior to data collection, ethics approval was obtained from the University of Luxembourg Ethics Review Panel (ERP 20–061-C I-SHARE-Lux), and participants gave consent electronically prior to participation.

Participants met the following inclusion criteria: a) aged 18 years or older, b) residing in Luxembourg and c) fluency in one of the survey languages (French, German, English, Portuguese). All individuals who met the inclusion criteria and had access to the advertisements were invited to participate. No financial compensation or incentive was offered.

The questionnaire was developed collaboratively by the I-SHARE consortium [15]. The same survey instrument was implemented in all countries. In addition, questions could be added for country-specific reasons.

The Luxembourgish versions of the instrument were translated from the English original version into French, German and Portuguese. Participants completed the questionnaire using OpenDataKit (University of Washington, USA) in 10–30 min. To complete the survey, participants did not have to respond to all questions, only items associated with skip patterns were mandatory.

Participants were asked to respond to questions concerning sexual behavior and substance use, considering the three months before COVID-19 measures were implemented (pre-COVID-19 measures) and while COVID-19 lockdown measures were in place (during-COVID-19 measures). The sexual behavior questions encompassed: masturbation, sexual intercourse with a steady and/or casual partner, sexting, cybersex and general sexual satisfaction. Sexual satisfaction was addressed with the question “how satisfied were you with your sex life?”, and sexual problems with the question: “how often have you or your sexual partner experienced sexual problems?”, using 4-point Likert scales (0 = not at all satisfied; 3 = very satisfied, and 0 = never; 3 = often, respectively). We also assessed potential changes in substance use frequency between pre- and during-COVID-19 measures concerning alcohol, tobacco, cannabis, cocaine, heroin, and methamphetamine. With respect to mental health, six items assessed emotional states (frustrated, confused, afraid), and cognitive and behavioral changes (obsessive compulsive hand washing, worrying about COVID-19). Response options were: no, neither agree nor disagree, yes. One question enquired about overall mental health perception (good or poor). Questions addressed the present time but also asked if the emotional state or behavior had changed since the COVID-19 measures.

We also included sociodemographic variables (age, sex, education level, number of children) and items on sexuality and relationship status (sexual orientation; general long-term partner variables, i.e. cuddling; condom use with casual and/or long-term partners during the specified time period).

### Statistical Analyses

Statistical analyses were conducted using SPSS 26. All participants with valid responses were included. As not all respondents were required to answer all questions, the number of respondents for each question varied.

First, we performed frequency analysis to describe the sample characteristics and the changes on sexual behaviors during the COVID-19 restriction measures in Luxembourg. Second, we performed a McNemar test to assess changes in sexual satisfaction and sexual problems before and during COVID-19-related restrictions. Lastly, to investigate the factors associated with sexual satisfaction during COVID-19 restrictions, we used a Binary Logistic Regression Model including as predictors demographic characteristics, sexual behaviors and mental health variables. In this step, the variable “sexual satisfaction” was recoded as a binary variable (0 = not at all/not very satisfied and 1 = somewhat/very satisfied).

## Results

A total of 557 individuals participated in this study, of who 196 (35.2%) identified their gender as male, 347 (62.3%) as female and 13 (2.3%) as “other”, “neither” or “both” genders. Regarding sexual orientation, 429 (77%) reported being heterosexual, 56 (10.1%) bisexual, 30 (3.6%) gay, 13(2.3%) asexual, 12 (2.2%) pansexual, 6 (1.1%) lesbian, 1 (0.2%) queer, 11 (2%) were questioning or unsure and 6 (1.1%) reported “other” sexual orientation. Regarding couple relationship status, 385 (69.1%) of the participants had a steady partner in the three months before COVID-19. Other demographic characteristics are described in Table 1.

**Table 1:** Demographic characteristics of participants

	N	%
Age (*N* = 557)		
Mean = 35.9 years; SD = 14.4		
Sex (*N* = 557)		
Man	198	35.5
Woman	358	64.3
Other	1	0.2
Gender (*N* = 556)		
Man	196	35.2
Woman	347	62.3
Neither	5	0.9
Other	3	0.5
Both	5	0.9
Relationship Status (*N* = 552)		
Single	172	31.1
Informal	178	32.2
Formal	174	31.5
Separated/Divorced/Widowed/Other	28	5.1
Schooling (*N* = 549)		
Primary school	14	2.5
Secondary school	160	28.7
Tertiary school	375	67.3
Religion (*N* = 555)		
Buddhist	1	0.2
Hindu	5	0.9
Jewish	2	0.4
Muslim	9	1.6
No religion	264	47.4
Orthodox	23	4.1
Protestant	16	2.9
Roman Catholic	218	39.1
Other	17	3.1
Accommodation (*n* = 555)		
Flat in flat block	219	39.3
House	282	50.6
Residential/retirement home	1	0.2
Room(s) in shared house	28	5
Student accommodation	19	3.4
Other	6	1.1
Household Income (*n* = 538)		
Prefer not to say	82	15.2
0 – 1250 Euros	11	2
1250 – 2000 Euros	23	4.3
2000 – 4000 Euros	31	5.8
6000 – 8000 Euros	84	15.6
8000 – 12,500 Euros	13	2.4
> 12,500 Euros	37	6.9

The majority of participants (*n* = 512, 92.2%) reported following the COVID-19 measures strictly or very strictly. Regarding COVID-19 testing, 472 (84.7%) of participants reported a negative COVID-19 test result. Forty-three participants (7.7%) had tested positive COVID-19 test at least once. Self-reported frequencies of sexual behaviors, sexual problems and sexual satisfaction during the pandemic restrictions are shown in Table 2. The majority of respondents reported no changes in most of their sexual behaviors compared to before the introduction of social distancing measures. For women, behaviors that changed the most were: cuddling with 28% reporting an increase and 25% a decrease, sexual activity with a steady partner with16% reporting an increase and 39.4% a decrease, and self-masturbation with almost identical percentages reporting an increase (20.3%) or decrease (20.6%). For men, smaller changes were observed. Among the sexual behaviors that changed the most were: cuddling (increase reported by 20.9%, and decrease reported by 16.4%), sexual activity with a steady partner (increase reported by 11.8%, decrease reported by 28.2%), masturbation (increase reported by 29%, decreased reported by 10.2%) and watching pornography (increase reported by 29.3%, decrease reported by 7.1%).

**Table 2:** Sexual behaviors, sexual problems and sexual satisfaction during COVID-19 restriction measures in Luxembourg

		All Participants		Women (*n* = 347)		Men (*n* = 196)		Other (*n* = 13)^a^	
		n	%	n	%	n	%	n	%
How satisfied were you with your sex life?		*n* = 531		*n* = 333		*n* = 186		*n* = 11	
	Not at all	94	17.7	48	14.4	44	23.7	2	18.2
	Not very	153	28.8	100	30	50	26.9	3	27.3
	Somewhat	183	34.5	118	35.4	59	31.7	5	45.5
	Very	101	19.0	67	20.1	33	17.7	1	9.1
How often have you or your partner experienced sexual problems?^b^		*n* = 325		*n* = 214		*n* = 111		*n* = 0	
	Never	128	39.4	78	36.4	50	45	0	0
	Once	24	7.4	19	8.9	5	4.5	0	0
	Sometimes	112	34.5	75	35	37	33.3	0	0
	Often	61	18.8	42	19.4	19	17.1	0	0
Cuddles with partner		*n* = 335		*n* = 216		*n* = 110		*n* = 9	
	Decreased	75	22.4	54	25	18	16.4	3	33.3
	The same	174	51.9	100	46.3	69	62.7	5	55.6
	Increased	86	25.7	62	28.7	23	20.9	1	11.1
Sexual activities with steady partner		*n* = 332		*n* = 213		*n* = 110		*n* = 9	
	Decreased	119	35.8	84	39.4	31	28.2	4	44.4
	The same	164	49.4	94	44.1	66	60.0	4	44.4
	Increased	49	14.8	35	16.4	13	11.8	1	11.1
Condom use with steady partner		*n* = 328		*n* = 210		*n* = 109		*n* = 9	
	Decreased	25	7.6	16	7.6	7	6.4	2	22.2
	The same	299	91.2	191	91	101	92.7	7	77.8
	Increased	4	1.2	3	1.4	1	0.9	0	0
Self-masturbation		*n* = 522		*n* = 325		*n* = 186		*n* = 11	
	Decreased	89	17	67	20.6	19	10.2	3	27.3
	The same	310	59.4	192	59.1	113	60.8	5	45.5
	Increased	123	23.6	66	20.3	54	29	3	27.3
Having sex with casual partner^c^		*n* = 509		*n* = 317		*n* = 183		*n* = 9	
	Decreased	73	14.3	34	10.7	37	20.2	2	22.2
	The same	401	78.8	262	82.6	132	72.1	7	77.8
	Increased	35	6.9	21	6.6	14	7.7	0	0
Condom use with a casual partner		*n* = 104		*n* = 46		*n* = 56		*n* = 2	
	Decreased	12	11.5	8	17.4	4	7.1	0	0
	The same	81	77.9	34	73.9	45	80.4	2	100
	Increased	11	10.6	4	8.7	7	12.5	0	0
Exchanging naked/semi-naked pictures, audio or videos with partner		*n* = 506		*n* = 319		*n* = 178		*n* = 9	
	Decreased	39	7.7	26	8.2	12	6.7	1	11.1
	The same	408	80.6	263	82.4	138	77.5	7	77.8
	Increased	59	11.7	30	9.4	28	15.7	1	11.1
Watching pornography videos		*n* = 515		*n* = 320		*n* = 184		*n* = 11	
	Decreased	56	10.9	40	12.5	13	7.1	3	27.3
	The same	365	70.9	240	75	117	63.6	8	72.7
	Increased	94	18.3	40	12.5	54	29.3	0	0
Performed/watched sexual acts before a webcam		*n* = 501		*n* = 315		*n* = 177		*n* = 9	
	Decreased	18	3.6	13	4.1	5	2.8	0	0
	The same	465	92.8	297	94.3	160	90.4	8	88.9
	Increased	18	3.6	5	1.6	12	6.8	1	11.1

^a^Other = participants that responded neither or other or both to the question “Which of the following do you identify as?”

^b^no participants under the category “Other” responded to this question

^c^The same: can indicate either the maintenance of having sex with casual partners or not engaging in such behavior

Regarding mental health, 42% (*n* = 235) reported poor overall mental health during COVID-19 restrictions. Participants also reported high rates of frustration because of these restrictions, as well as other negative feelings, which are summarized in Table 3.

**Table 3:** Perceptions on COVID-19 and mental health

	n	%
How would you rate your overall mental health?		
Good	320	57.5
Poor	235	42.2
I feel frustrated because of COVID-19 restrictions		
No	64	11.5
Nor agree or disagree	69	12.4
Yes	422	75.8
I am confused about what I can or cannot do due to COVID-19		
No	199	35.7
Nor agree or disagree	90	16.5
Yes	266	47.8
I am afraid to acquire COVID-19		
No	154	27.7
Nor agree or disagree	122	21.9
Yes	280	50.5
I experience obsessive or compulsive behaviors with regards to hand washing		
No	334	60.0
Nor agree or disagree	128	23
Yes	94	16.9
I cannot stop thinking about the COVID-19 epidemic		
No	278	49.9
Nor agree or disagree	120	21.5
Yes	158	28.4

Sexual problems experienced in the partnership increased significantly during COVID-19 measures, from 42.5% (*n* = 138) to 53.2% (*n* = 173); *p* < 0.001. Sexual satisfaction decreased significantly during social distancing measures from 71.1% (*n* = 377) to 53.6% (*n* = 284); *p* < 0.001.

The factors associated with increased sexual satisfaction during COVID-19-related measures were assessed with a Binary Logistic Regression Model (Table 4). The results summarized in Table 4 show that participants who were in a steady relationship before COVID-19 restrictions had higher odds of reporting increased sexual satisfaction (OR = 2.4, 95% CI = 1.5 – 3.7, *p* < 0.00). Regarding sexual behaviors, participants who reported an increase in exchanging sex-content messages with a partner had higher odds of reporting increased sexual satisfaction (OR = 2.9, 95% CI = 1.0 – 8.3, p = 0.04). Those who reported a decrease (OR = 2.2, 95% CI = 1 – 4.9, *p* = 0.03) or no changes (OR = 2.8, 95% CI = 1.5 – 5.2, *p* = 0.001) in self-masturbation, had higher odds of reporting increased sexual satisfaction.

**Table 4:** Binary Logistic Regression Model for sexual satisfaction during COVID-19 measures

	n	Satisfied % (n)	OR (95% CI)	*p*-value
Age				
	480		.98 (.96 – 1.00)	.07
Sex^a^				
Men	170	51.2% (87)	.85 (.55 – 1.33)	.49
Women	310	56.1% (174)		
Number of children				
	480		1.10 (.85– 1.42)	.45
Schooling				
Primary school	10	70% (7)	3.03 (.69 – 13.27)	.14
Secondary school	130	60.8% (79)	1.58 (.99 – 2.52)	.05
Tertiary school	341	51.5% (175)		.06
Steady partner during COVID-19				
Yes	347	61.4% (213)	2.41 (1.54 – 3.78)	**.00**
No	133	36.1% (48)		
How often did you have a drink containing alcohol (during COVID)?				
Decreased	139	48.2% (67)	1.23 (.69 – 2.20)	.48
The same	235	62.1% (146)	1.79 (1.05 – 3.04)	**.03**
Increased	106	45.3% (48)		.07
How would you rate your overall mental health?				
Good	274	62.4% (171)	1.93 (1.26 – 2.96)	**.002**
Poor	206	43.7% (90)		
I feel frustrated because of COVID-19 restrictions				
No	56	51.8% (29)	.66 (.33 – 1.32)	.24
Nor agree or disagree	58	69% (40)	1.49 (.74 – 2.97)	.26
Yes	366	52.5% (192)		.20
I am confused about what I can or cannot do due to COVID-19				
No	173	57.2% (99)	1.38 (.83 – 2.28)	.20
Nor agree or disagree	76	61.8% (47)	1.47 (.79 – 2.75)	.20
Yes	231	49.8% (11)		.31
I am afraid to acquire COVID-19				
No	134	52.2% (7)	.84 (.49 – 1.41)	.51
Nor agree or disagree	105	62.9% (66)	1.28 (.74 – 2.21)	.37
Yes	241	51.9% (125)		.38
I experience obsessive or compulsive behaviors with regards to hand washing				
No	292	56.5% (165)	.91 (.50 – 1.66)	.77
Nor agree or disagree	108	50% (54)	.69 (.36 – 1.35)	.28
Yes	80	52.5% (42)		.49
I cannot stop thinking about the COVID-19 epidemic				
No	241	57.3% (138)	1.16 (.66 – 2.03)	.58
Nor agree or disagree	98	58.2% (57)	1.21 (.66 – 2.21)	.52
Yes	141	46.8% (66)		.79
Watching pornography videos				
Decreased	52	44.2% (23)	1.18 (.44 – 3.16)	.73
The same	339	60.8% (206)	1.55 (.77 – 3.12)	.21
Increased	89	36% (32)		.42
Exchanging naked/semi = naked pictures, audios or videos with partner				
Increased	53	50.9% (27)	2.93 (1.02 – 8.35)	**.044**
The same	390	56.7% (221)	2.09 (.89 – 4.88)	.08
Decreased	37	35.1% (13)		.11
Performed/watched sexual acts before a webcam				
Increased	18	55.6% (10)	4.28 (.86 – 21.25)	.07
The same	444	55% (244)	1.11 (.33 – 3.70)	.85
Decreased	18	38.9% (7)		.08
Self-masturbation				
Decreased	82	52.4% (43)	2.26 (1.04 – 4.93)	**.039**
The same	285	62.8% (179)	2.81 (1.50 – 5.26)	**.001**
Increased	113	34.5% (39)		**.005**

Dependent variable: sexual satisfaction, recoded as 0 = not at all/not very satisfied and 1 = somewhat/very satisfied

^a^Respondent assigned as “other” did not reply to all items in this regression model and therefore was not included in the analysis

Good mental health was associated with an increase in sexual satisfaction. Participants who reported “good” general mental health had higher odds of increased sexual satisfaction than those who indicated “poor” mental health (OR = 1.9, 95% CI = 1.2 – 2.9, p = 0.002). Concerning alcohol consumption, no changes in self-reported alcohol intake were associated with higher odds for increased sexual satisfaction (OR = 1.7, 95% CI = 1.0 – 3.0, *p* = 0.03).

## Discussion

The present study examined the impact of measures imposed by the Luxembourgish government to fight the COVID-19 pandemic on self-reported sexual behaviors, substance use and mental health.

We found a decrease in sex frequency during COVID-19 compared to the period before the introduction of the COVID-19 measures. The proportion of respondents reporting a decrease in sexual activities was higher in those with steady partners (35.8%) compared to those with casual partners (14.3%). This decrease was larger than the one found in a study comparing individuals from the English and Spanish populations [7], but smaller than the decrease found in a multi-country study with 30 different countries [19]. The reasons for these differences between studies are unclear. Nevertheless, any decrease in sexual activity could have overall health implications, as a decrease in sexual intercourse has been reported to be associated with an overall decline in well-being [11].

A large proportion of the present sample (46.5%) reported low sexual satisfaction. Similar findings were also found in other I-SHARE countries where, overall, 39.6% reported low sexual satisfaction [19]. This demonstrates the need for health professionals to address sexual and reproductive health issues during the COVID-19 pandemic and the importance of sexual health for general health and wellbeing.

Our data indicate that being in a relationship before the implementation of the COVID-19 measures increased the chances of sexual satisfaction during COVID-19. This is in line with the literature, which found that people in steady relationships during the COVID-19 measures were more sexually active and also more satisfied [19]. This corroborates the evidence on the association between sexual satisfaction and more frequent sexual activity [20, 21]. In addition, this is in accordance with the finding that an increase in self-masturbation was associated with higher odds of increased sexual dissatisfaction. In this context, it is plausible that self-masturbation might have been used as a coping strategy for some. In the present sample, we found a 18% increase in the frequency of masturbation, which is almost twice the percentage reported by Ibarra et al. [7], but a very similar prevalence of no change in autoerotism when compared to an Italian study (61.2% in Italy, 59.9% in Luxembourg) [11].

Findings on the relationship between masturbatory behavior and sexual activity and satisfaction are inconsistent. Masturbation offers the possibility of sexual pleasure independent of a partner’s availability and sexual health. For women, masturbation seems to be related to more consistent orgasms compared with partnered sex [22–24]. Nonetheless, weak or negative associations between solitary and partnered sexual activity or satisfaction have been found [25, 26].

With regards to sexual problems, our findings show an increase in sexual problems in those in partnership (either oneself or of the partner) during the pandemic. This result is in line with other studies that have addressed this question. Since the beginning of the pandemic, an increase in sexual problems has been found in a range of populations in different countries, for instance in COVID-19 positive women [27] and in uninfected pregnant women in Turkey [28], in women in the U.S. [29] and in men and women in Egypt [30].

Our data suggest that people who increased sexting (exchange of sex-content messages such as naked/semi-naked pictures, audios or videos with a partner) had higher odds of reporting sexual satisfaction. Sexting and cybersex might have acted as a tool for different sexual activities in a person or couple’s sexual repertoire [31]. Our results are in line both with other studies conducted during the COVID-19 pandemic that found the use of digital means for sexual communication a way to maintain oneself sexually active [7] and with the literature on sexting and relationship satisfaction that found higher relationship satisfaction in people who engaged in sexting [32–34].

With respect to mental health, the ability to adapt to the new pandemic context benefitted levels of sexual satisfaction. This becomes clear when observing that respondents who reported good general mental health presented higher scores of sexual satisfaction. This is in line with the literature, that found a higher risk of developing anxiety and depression among those who were not sexually active during the lockdown [13]. Other individual and couple protective factors to maintain mental health during the pandemic included problem-solving skills, positive reappraisal, self-efficacy, intimate partnership, social integration and mindfulness [35–37]. Similarly, to the adaptation findings regarding mental health, participants who reported no change in alcohol consumption had higher odds of reporting sexual satisfaction. This can possibly be explained by better personal resources to adapt to the pandemic context, and not using alcohol as a coping strategy to deal with the COVID-19 measures. Alcohol consumption as a coping strategy has been identified in previous pandemic outbreaks, e.g. severe acute respiratory syndrome [38]. So far, during the COVID-19 pandemic, an increase in alcohol consumption has been reported both in the general population [39, 40] and among university students [41].

### Strengths and Limitations

This study has several limitations. First, the data collection was carried out 9 months after the Luxembourg COVID-19 outbreak. Taking into consideration that some questions concerned behaviors in the 3 months prior to the pandemic outbreak, a retrospective memory bias may have occurred. Second, our study was conducted online which can lead to selection bias (e.g., only people with internet access could take part). Third, our sample was recruited using a convenience approach, predominantly via social media platforms and invitations to key sexual health organizations of the country, which limits the generalizability of the study findings. To overcome such conditions, we employed a broad recruitment strategy [42], using different social media, traditional media, press release, partnering with key governmental and non-governmental institutions as well as invitations to participants of previous COVID19 studies who agreed to be contacted for further studies. Fourth, the results obtained do not offer the possibility to evaluate factors associated with changes on sexual satisfaction, but only those associated with satisfaction while COVID-19 restrictions were in place.

Despite these limitations, this study contributes to the literature on sexual behavior during COVID-19, and the results indicate the importance of continuing research to support policy and help care provision. From a research and policy perspective, longitudinal assessments of the population are needed to properly identify their health needs. On a care provision level, sexual and mental health professionals should be trained and updated to face the population’s new demands with reference to sexual behaviors and satisfaction during times of crises such as infectious disease outbreaks.

## Conclusions

The COVID-19 pandemic and the social distancing measures impacted sexual behaviors of the inhabitants of Luxembourg. Sexual and reproductive health care centers and other health professionals should take these results into consideration when providing care. Recommendations on the importance of sexual health for general wellbeing and behaviors associated with sexual satisfaction should be offered and possibilities to experience sexuality while reducing contamination risks be discussed.

## Data Availability

The datasets generated and/or analysed during the current study are not publicly available due to the agreement established by the I-SHARE international Consortium parties but are available from the corresponding author on reasonable request.

## References

[1] Haider N, Osman AY, Gadzekpo A, Akipede GO, Asogun D, Ansumana R (2020). Lockdown measures in response to COVID-19 in nine sub-Saharan African countries. BMJ Glob Heal.

[2] Luxembourg Research (2021). Luxembourg comes second in dealing with COVID-19.

[3] Luxembourg Research (2020). Large Scale Testing.

[4] Vögele C, Ortmann J, Lutz APC, Schulz A, van Dyck Z, D‘Ambrosio C (2021). The impact of social isolation and loneliness on mental health and well-being: the COVID-19 pandemic. Self and society in the Corona crisis.

[5] Schiavi MC, Spina V, Zullo MA, Colagiovanni V, Luffarelli P, Rago R (2020). Love in the Time of COVID-19: sexual function and quality of life analysis during the social distancing measures in a Group of Italian reproductive-age women. J Sex Med.

[6] Evans S, Mikocka-Walus A, Klas A, Olive L, Sciberras E, Karantzas G, et al. From “It Has Stopped Our Lives” to “Spending More Time Together Has Strengthened Bonds”: The Varied Experiences of Australian Families During COVID-19. Front Psychol. 2020;11. Available from: https://www.frontiersin.org/articles/10.3389/fpsyg.2020.588667/full 10.3389/fpsyg.2020.588667 PMC7606874 33192922

[7] Ibarra FP, Mehrad M, Di Mauro M, Peraza Godoy MF, Cruz EG, Nilforoushzadeh MA (2020). Impact of the COVID-19 pandemic on the sexual behavior of the population. The vision of the east and the west. Int Braz J Urol.

[8] Lehmiller JJ, Garcia JR, Gesselman AN, Mark KP (2021). Less sex, but more sexual diversity: changes in sexual behavior during the COVID-19 Coronavirus pandemic. Leis Sci.

[9] Insights P. Coronavirus update – June 18. Available from: https://www.pornhub.com/insights/coronavirus-update-june-18. Cited 2021 Sep 2

[10] Li W, Li G, Xin C, Wang Y, Yang S (2020). Challenges in the practice of sexual medicine in the time of COVID-19 in China. J Sex Med.

[11] Cito G, Micelli E, Cocci A, Polloni G, Russo GI, Coccia ME (2021). The impact of the COVID-19 quarantine on sexual life in Italy. Urology.

[12] Arafat SMY, Alradie-Mohamed A, Kar SK, Sharma P, Kabir R (2020). Does COVID-19 pandemic affect sexual behaviour? A cross-sectional, cross-national online survey. Psychiatry Res.

[13] Mollaioli D, Sansone A, Ciocca G, Limoncin E, Colonnello E, Di Lorenzo G (2021). Benefits of sexual activity on psychological, relational, and sexual health during the COVID-19 breakout. J Sex Med.

[14] Mitchell KR, Lewis R, O’Sullivan LF, Fortenberry JD. What is sexual wellbeing and why does it matter for public health? Lancet Public Heal. 2021 Aug;6(8):e608–13. Available from: https://linkinghub.elsevier.com/retrieve/pii/S2468266721000992 10.1016/S2468-2667(21)00099-2 PMC7616985 34166629

[15] Michielsen K, Larrson EC, Kågesten A, ErausquinGriffin JTS, Van De Velde S (2021). International Sexual Health and REproductive health (I-SHARE) survey during COVID-19: study protocol for online national surveys and global comparative analyses. Sex Transm Infect.

[16] Luxembourg Government (2020). A small but open society.

[17] Comission E (2021). Eurostat - population density.

[18] Crisis24.Garda. Luxembourg: Authorities maintain COVID-19-related measures nationwide as of Jan. 15 /update 10. 2021.

[19] Erausquin JT, Tan RKJ, Uhlich M, Francis JM, Kumar N, Campbell L, et al. The International Sexual Health And Reproductive Health Survey (I-SHARE-1): A Multi-Country Analysis of Adults from 30 Countries Prior to and During the Initial COVID-19 Wave. medRxiv. 2021;2021.09.18.21263630. Available from: http://medrxiv.org/content/early/2021/10/19/2021.09.18.21263630.abstract

[20] Blair KL, Pukall CF (2014). Can less be more? Comparing duration vs. frequency of sexual encounters in same-sex and mixed-sex relationships. Can J Hum Sex.

[21] Higgins JA, Mullinax M, Trussell J, Davidson JK, Moore NB (2011). Sexual satisfaction and sexual health among University students in the United States. Am J Public Health.

[22] Dekker A, Schmidt G (2003). Patterns of masturbatory behaviour. J Psychol Human Sex.

[23] Howard JR, O’Neill S, Travers C (2006). Factors affecting sexuality in older Australian women: sexual interest, sexual arousal, relationships and sexual distress in older Australian women. Climacteric.

[24] Hinchliff S, Tetley J, Lee D, Nazroo J (2018). Older adults’ experiences of sexual difficulties: qualitative findings from the English Longitudinal Study on Ageing (ELSA). J Sex Res.

[25] Brody S, Costa RM (2009). Original Research—Anatomy/Physiology: satisfaction (sexual, life, relationship, and mental health) is associated directly with penile–vaginal intercourse, but inversely with other sexual behavior frequencies. J Sex Med.

[26] Rowland DL, Kolba TN, McNabney SM, Uribe D, Hevesi K (2020). Why and how women masturbate, and the relationship to orgasmic response. J Sex Marital Ther.

[27] Kaya Y, Kaya C, Tahta T, Kartal T, Tokgöz VY (2021). Examination of the effect of COVID-19 on sexual dysfunction in women. Int J Clin Pract.

[28] Karakas LA, Azemi A, Simsek SY, Akilli H, Esin S (2021). Risk factors for sexual dysfunction in pregnant women during the COVID-19 pandemic. Int J Gynecol Obstet.

[29] Bhambhvani HP, Chen T, Kasman AM, Wilson-King G, Enemchukwu E, Eisenberg ML (2021). Female sexual function during the COVID-19 pandemic in the United States. Sex Med.

[30] Omar SS, Dawood W, Eid N, Eldeeb D, Munir A, Arafat W (2021). Psychological and sexual health during the COVID-19 pandemic in Egypt: are women suffering more?. Sex Med.

[31] Courtice EL, Shaughnessy K (2017). Technology-mediated sexual interaction and relationships: a systematic review of the literature. Sex Relatsh Ther.

[32] Currin JM, Jayne CN, Hammer TR, Brim T, Hubach RD (2016). Explicitly pressing send: impact of sexting on relationship satisfaction. Am J Fam Ther.

[33] McDaniel BT, Drouin M (2015). sexting among married couples: who is doing it, and are they more satisfied?. Cyberpsychol Behav Soc Netw.

[34] Parker TS, Blackburn KM, Perry MS, Hawks JM (2013). Sexting as an Intervention: Relationship Satisfaction and Motivation Considerations. Am J Fam Ther.

[35] de Ferreira FO, Lopes-Silva JB, Siquara GM, Manfroi EC, de Freitas PM (2021). Coping in the COVID-19 pandemia: how different resources and strategies can be risk or protective factors to mental health in the Brazilian population. Heal Psychol Behav Med.

[36] Preetz R, Filser A, Brömmelhaus A, Baalmann T, Feldhaus M (2021). Longitudinal changes in life satisfaction and mental health in emerging adulthood during the COVID-19 pandemic. Risk and protective factors. Emerg Adulthood.

[37] Racine S, Miller A, Mehak A, Trolio V (2022). Examining risk and protective factors for psychological health during the COVID-19 pandemic. Anxiety, Stress Coping.

[38] Wu P, Liu X, Fang Y, Fan B, Fuller CJ, Guan Z (2008). Alcohol abuse/dependence symptoms among hospital employees exposed to a SARS Outbreak: Table 1. Alcohol Alcohol.

[39] Vanderbruggen N, Matthys F, Van Laere S, Zeeuws D, Santermans L, Van den Ameele S (2020). Self-reported alcohol, tobacco, and cannabis use during COVID-19 lockdown measures: results from a web-based survey. Eur Addict Res.

[40] Malta DC, Szwarcwald CL, Barros MB de A, Gomes CS, Machado ÍE, Souza Júnior PRB de, et al. A pandemia da COVID-19 e as mudanças no estilo de vida dos brasileiros adultos: um estudo transversal, 2020. Epidemiol e Serviços Saúde. 2020;29(4). Available from: http://www.scielo.br/scielo.php?script=sci_arttext&pid=S2237-96222020000400315&tlng=pt 10.1590/S1679-49742020000400026 32997069

[41] Romero-Blanco C, Rodríguez-Almagro J, Onieva-Zafra MD, Parra-Fernández ML, del Prado-Laguna MC, Hernández-Martínez A (2020). Physical activity and sedentary lifestyle in university students: changes during confinement due to the COVID-19 pandemic. Int J Environ Res Public Health.

[42] Hlatshwako TG, Shah SJ, Kosana P, Adebayo E, Hendriks J, Larsson EC (2021). Online health survey research during COVID-19. Lancet Digit Heal.

